# Aboveground herbivory does not affect mycorrhiza-dependent nitrogen acquisition from soil but inhibits mycorrhizal network-mediated nitrogen interplant transfer in maize

**DOI:** 10.3389/fpls.2022.1080416

**Published:** 2022-12-14

**Authors:** Chenling He, Yibin Lin, Yifang Zhang, Lu Tong, Yuanxing Ding, Min Yao, Qian Liu, Rensen Zeng, Dongmei Chen, Yuanyuan Song

**Affiliations:** ^1^ State Key Laboratory of Ecological Pest Control for Fujian and Taiwan Crops, Breeding and Multiple Utilization of Crops, College of Agriculture, Fujian Agriculture and Forestry University, Fuzhou, China; ^2^ Key Laboratory of Ministry of Education for Genetics, Breeding and Multiple Utilization of Crops, College of Agriculture, Fujian Agriculture and Forestry University, Fuzhou, China; ^3^ Institute of Chemical Ecology and Crop Resistance, Fujian Agriculture and Forestry University, Fuzhou, China

**Keywords:** mycorrhiza, nitrogen, *Zea mays*, common mycorrhizal networks, insect herbivory

## Abstract

Arbuscular mycorrhizal fungi (AMF) are considered biofertilizers for sustainable agriculture due to their ability to facilitate plant uptake of important mineral elements, such as nitrogen (N). However, plant mycorrhiza-dependent N uptake and interplant transfer may be highly context-dependent, and whether it is affected by aboveground herbivory remains largely unknown. Here, we used ^15^N labeling and tracking to examine the effect of aboveground insect herbivory by *Spodoptera frugiperda* on mycorrhiza-dependent N uptake in maize (*Zea mays* L.). To minimize consumption differences and ^15^N loss due to insect chewing, insect herbivory was simulated by mechanical wounding and oral secretion of *S. frugiperda* larvae. Inoculation with *Rhizophagus irregularis* (*Rir*) significantly improved maize growth, and N/P uptake. The ^15^N labeling experiment showed that maize plants absorbed N from soils *via* the extraradical mycelium of mycorrhizal fungi and from neighboring plants transferred by common mycorrhizal networks (CMNs). Simulated aboveground leaf herbivory did not affect mycorrhiza-mediated N acquisition from soil. However, CMN-mediated N transfer from neighboring plants was blocked by leaf simulated herbivory. Our findings suggest that aboveground herbivory inhibits CMN-mediated N transfer between plants but does not affect N acquisition from soil solutions *via* extraradical mycorrhizal mycelium.

## 1 Introduction

Nitrogen (N) is one of the most important nutrients that limit plant growth and crop yield (Crawford and Glass, 1998). Beneficial microbes, such as mycorrhizal fungi, rhizobial bacteria, and plant growth promoting bacteria (PGPR), play a key role in the acquisition of plant nutrients in natural ecosystems (Etesami and Adl, 2020; Jaiswal et al., 2021; Ma et al., 2021). Arbuscular mycorrhizal fungi (AMF) belonging to the subphylum Glomeromycotina can establish mutually beneficial relationships with most land plants (including the most important crops) to form arbuscular mycorrhizae (Choi et al., 2018), in which AMF supply plants with multiple nutrients including N, and in return gain photosynthetic products from plants in the form of sugars (Ge et al., 2008; Boldt et al., 2011; An et al., 2019) and lipids (Jiang et al., 2017; Wang et al., 2017). The inorganic nutrients obtained through AMF promote plant growth and development (Smith et al., 2003; Smith et al., 2004; Yang et al., 2012; Wipf et al., 2019). Studies have shown that 1/3 of plant root N can be supplied by symbiotic AMF in the form of amino acids (Govindarajulu et al., 2005). Although the role of N in mycorrhizal symbiosis is not as clear as that of P, it has been demonstrated that AMF are able to absorb $NH_{4}^{+}$ (Frey and Schüepp, 1993), $NO_{3}^{-}$ (Tobar et al., 1994), and organic N (such as amino acids) (Cliquet et al., 1997). In addition, transporter genes involved in mycorrhizal N uptake have also been identified in many important crops, such as *GmAMT3;1*, *GmAMT4;4*, *GmAMT4;1* and *GmAMT1;4* in soybean (*Glycine max* L.) (Kobae et al., 2010), *OsAMT3;1* in rice (*Oryza sativa* L.) (Pérez-Tienda et al., 2011), *SbAMT3;1* in sorghum (*Sorghum bicolor* L.) (Koegel et al., 2013), and *LeAMT4* and *LeAMT5* in tomato (*Solanum lycopersicum* L.) (Ruzicka et al., 2012).

For the purpose of exploring nutrients, extraradical mycorrhizal mycelia (ERM) expand and grow, and by means of infection of neighboring plants and hyphal fusion, common mycorrhizal networks (CMNs) are formed to connect multiple plants, strengthening the connections, communications and especially the transport and exchange of nutrients among plants (Whiteside et al., 2019). Typically, CMNs play a crucial role in the distribution of nutrients from a shared resource pool among different plants. Plant-to-plant N transfer mediated by CMNs has important implications for improving yields in agricultural and forest systems (He et al., 2003; Hodge and Storer, 2015). In many cases, N is transferred from N-fixing or N-rich mycorrhizal plants to N-poor or non-N-fixing mycorrhizal plants through CMNs, for example, from soybean to maize (*Zea mays* L.) (Wang et al., 2016), from alfalfa (*Medicago sativa* L.) to maize (Zhang et al., 2020), and from faba bean (*Vicia faba* L.) to wheat (*Triticum turgidum* L.) (Wahbi et al., 2016) in intercropping systems. However, some studies have also shown that CMN-mediated N transfer from non-N-fixing to N-fixing mycorrhizal plants also occurs, and that such transfer is bidirectional (Johansen and Jensen, 1996; He et al., 2004). Therefore, CMN-mediated interplant N transfer may be widespread, and plant-derived N transfer can be viewed as a complement to plant mycorrhiza-dependent N uptake.

The improvement of plant mineral nutrition by ERM or CMNs has been widely recognized (Wipf et al., 2019), however, in natural environments, mycorrhizal plants often interact with other biotic or abiotic factors (Almario et al., 2022). Therefore, a comprehensive understanding of how mycorrhizal plants interact with other biotic or abiotic factors is particularly important for understanding the improvement of crop mineral nutrition by ERM or CMNs. Aboveground herbivory is one of the main factors limiting agricultural production (Oerke, 2006). Herbivory usually impairs carbon (C) fixation in plants by directly depleting plant photosynthetic tissue or indirectly inhibiting photosynthesis (Nabity et al., 2013), and this may affect nutrient exchange and transfer between plants and AMF. Currently, researchers are interested in how aboveground herbivory affects nutrient exchange between plants and AMF. It has been reported that the aboveground herbivory by the generalist insect *Helicoverpa punctigera* inhibits plant mycorrhiza-dependent P uptake (Frew, 2021); Charters et al. revealed that host plants transferred less C to their symbiotic fungal partners upon aphid attack, whereas the P supply from AMF to host plants was unaffected (Charters et al., 2020). This report demonstrated that nutrient exchange between AMF and host plants can be quantified in the presence of exogenous sap-sucking herbivores (Merckx and Gomes, 2020). In addition, AMF colonization and AMF-mediated plant growth responses are not affected by aphid feeding or by N or P uptake (Charters et al., 2022). The different effects of aboveground herbivory on mycorrhizae-mediated mineral nutrient uptake may be related to herbivorous types, AMF or plant species. Therefore, mycorrhiza-dependent mineral nutrient uptake may have a high context dependency (Pozo et al., 2015).

Maize is an important economic crop as well as a model plant for studying mycorrhizal symbiosis. Insect herbivores, especially invasive fall-armyworm (*Spodoptera frugiperda*) in Africa and Asia, are likely to be a major constraint on maize production in these regions (Xiao et al., 2020; Paredes-Sánchez et al., 2021; Prasanna et al., 2022). In this study, we systematically evaluated N acquisition from soil by ERM and N transfer from neighboring plants *via* CMNs. Aboveground herbivory did not affect mycorrhiza-dependent N acquisition from soil but inhibited CMN-mediated N interplant transfer in maize plants.

## 2 Results

### 2.1 Mycorrhizal symbiosis promotes plant growth and N accumulation in maize

**Figure 1:**
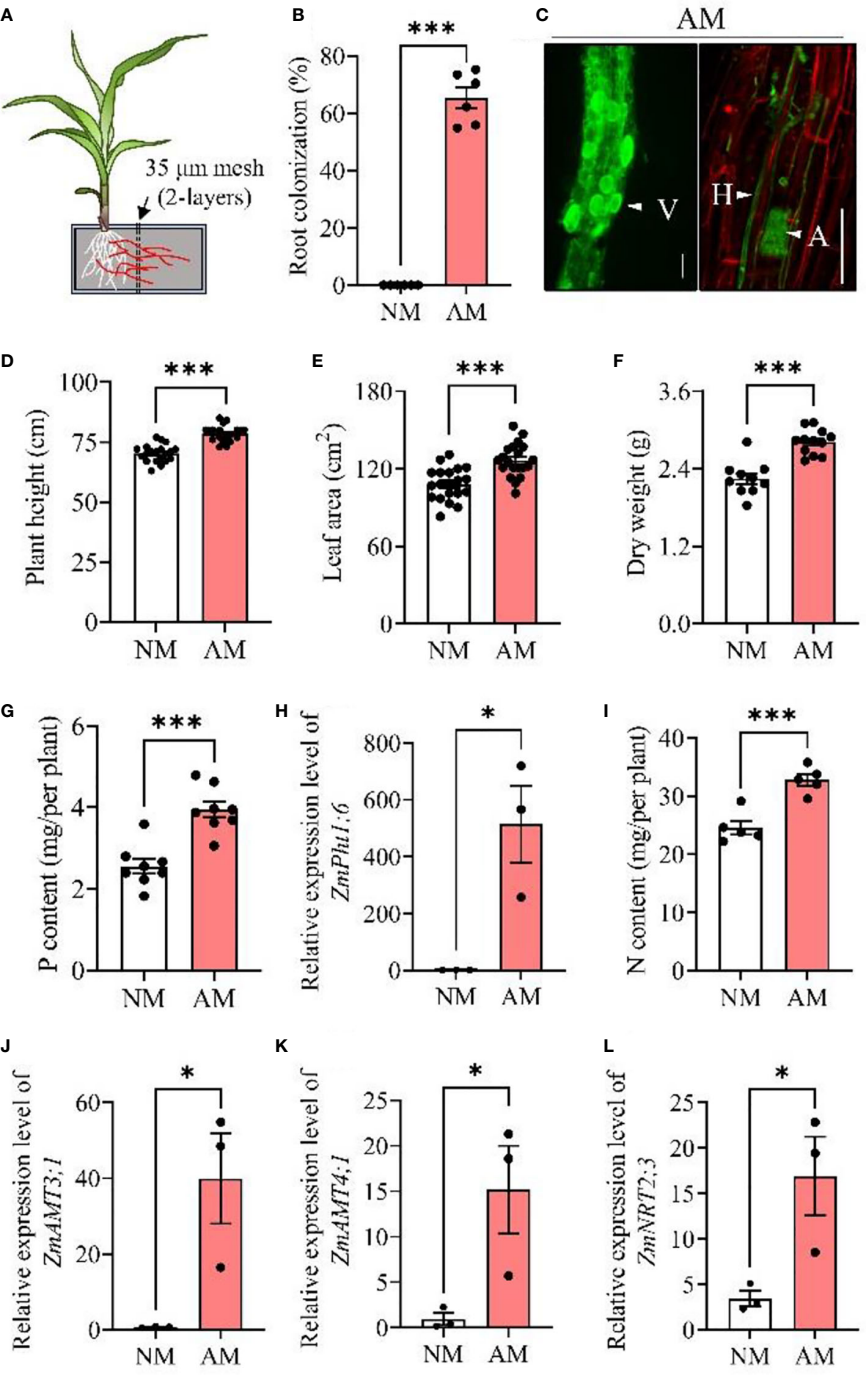
Mycorrhizal symbiosis promotes the growth and nutrient uptake of maize plants. Maize plants were inoculated with *Rhizophagus irregularis* (*Rir*) and cultivated in self-designed boxes **(A)**. Five weeks post-*Rir* inoculation, maize plant roots were harvested for the measurement of mycorrhizal root colonization rate **(B)** and mycorrhizal observation by wheat germ agglutinin (WGA) fluorescence staining **(C)** (bar scale: 25 μm). Plant height **(D)**, leaf area **(E)**, and dry weight **(F)** were measured, followed by the analysis of phosphorus content **(G)**, *ZmPht1;6* (a mycorrhizal symbiosis marker gene in maize) expression **(H)**, nitrogen content **(I)** and N transporter gene expressions **(J–L)**. For analysis of root colonization, n = 6; plant height and leaf area, n = 20; dry weight, n = 10; nitrogen content, n = 5; phosphorus content, n = 8; expression of *ZmPht1;6* and N transporter genes, n = 3. Data are means ± SE. Asterisks at the top of the bars indicate statistical significance (Student’s t-test, *P < 0.05; ***P <  0.001; ns, no significance). NM, non-mycorrhizal maize plants; AM, arbuscular mycorrhizal maize plants.

The mycorrhizal colonization rate in maize roots was measured five weeks post *Rhizophagus irregularis* (*Rir*) inoculation as illustrated in Experiment I (**Figure 1A**). The mycorrhizal symbiosis relationship was well developed, with colonization rate over 60% in maize roots (**Figure 1B**), and the typical structures of vesicles, hyphae and arbuscules were observed by staining the mycorrhizal roots (**Figure 1C**), showing successful mycorrhizal colonization in maize plants. Furthermore, mycorrhizal inoculation on maize plants led to increases in plant height by 11.9% (*P*< 0.001, **Figure 1D**), leaf area by 17.1% (*P*< 0.001, **Figure 1E**), and dry weight by 25.3% (*P*< 0.001, **Figure 1F**). Compared to non-mycorrhizal maize plants, P content in arbuscular mycorrhizal maize plants was significantly increased (**Figure 1G**), and the expression of mycorrhizal inducible phosphate transporter gene *ZmPht1;6* (a mycorrhizal symbiosis marker gene in maize (Yu et al., 2018)) was increased by 54.5% (*P<* 0.001, **Figure 1H**). In addition to P, N accumulation was also enhanced by 32.5% in mycorrhizal symbiosis plants (*P*< 0.001, **Figure 1I**), and the expression of three N transporter genes (including *ZmAMT3;1*, a recently reported mycorrhiza induced N transporter gene) was upregulated during mycorrhizal symbiosis (**Figures 1J-L**).

### 2.2 Maize plants can acquire N from soils *via* ERM

**Figure 2:**
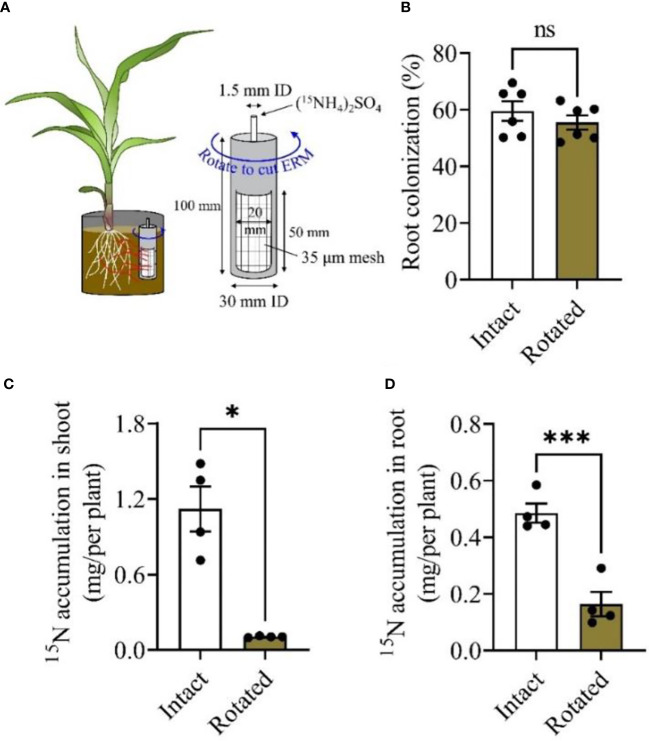
Mycorrhiza-mediated nitrogen uptake from soil by maize plants. Maize plants were inoculated with the mycorrhizal fungus *Rhizophagus irregularis* (*Rir*) and cultivated in the boxes with a PVC core in each box **(A)**. Five weeks post *Rir* inoculation, the PVC core was injected with (^15^NH_4_)_2_SO_4_ solution, and was then either rotated daily (for the purpose of damaging the mycelium) or non-rotated (intact for maintaining mycelial contact to labeling ^15^N). Plants were harvested to measure the root colonization rate **(B)** and the accumulation of shoot ^15^N **(C)** and root ^15^N **(D)** ten days after labeling N injection. For the analysis of root colonization, n = 6; ^15^N accumulation, n = 4. Data are the means ± SE of biological replicates. Asterisks indicate significant differences (Student’s t-test, *P < 0.05; ***P < 0.001; ns, no significance).

The exchange of nutrients between host plants and mycorrhizal fungi is considered the main benefit for the two symbiotic partners (Wipf et al., 2019). Therefore, we determined whether maize plants acquire N through mycorrhizal pathways similar to other plants described in previous studies (Frey and Schüepp, 1993; Tobar et al., 1994). As illustrated in Experiment II (**Figure 2A**), the PVC core was rotated daily to cut the extraradical mycelium (ERM) to block the ^15^N transfer from the PVC core to plant roots. Rotation treatment did not affect root colonization compared to nontreated (Intact) maize plants (**Figure 2B**). ^15^N accumulation in the shoots of maize plants with ERM into a PVC core (Intact) was significantly higher than that in the plants that did not obtain N from PVC core (Rotated) (**Figure 2C**; **S1A**), reaching a net ^15^N accumulation of 1.01 mg/per plant shoot. A consistent result was also observed in maize roots, in that the rotation treatment decreased ^15^N accumulation compared to Intact treatment (**Figure 2D**; **S1B**). These results further confirmed that maize plants acquired N through the mycorrhizal pathway *via* ERM from the soil habitat.

### 2.3 Aboveground herbivory does not affect maize N uptake from soil *via* ERM

**Figure 3:**
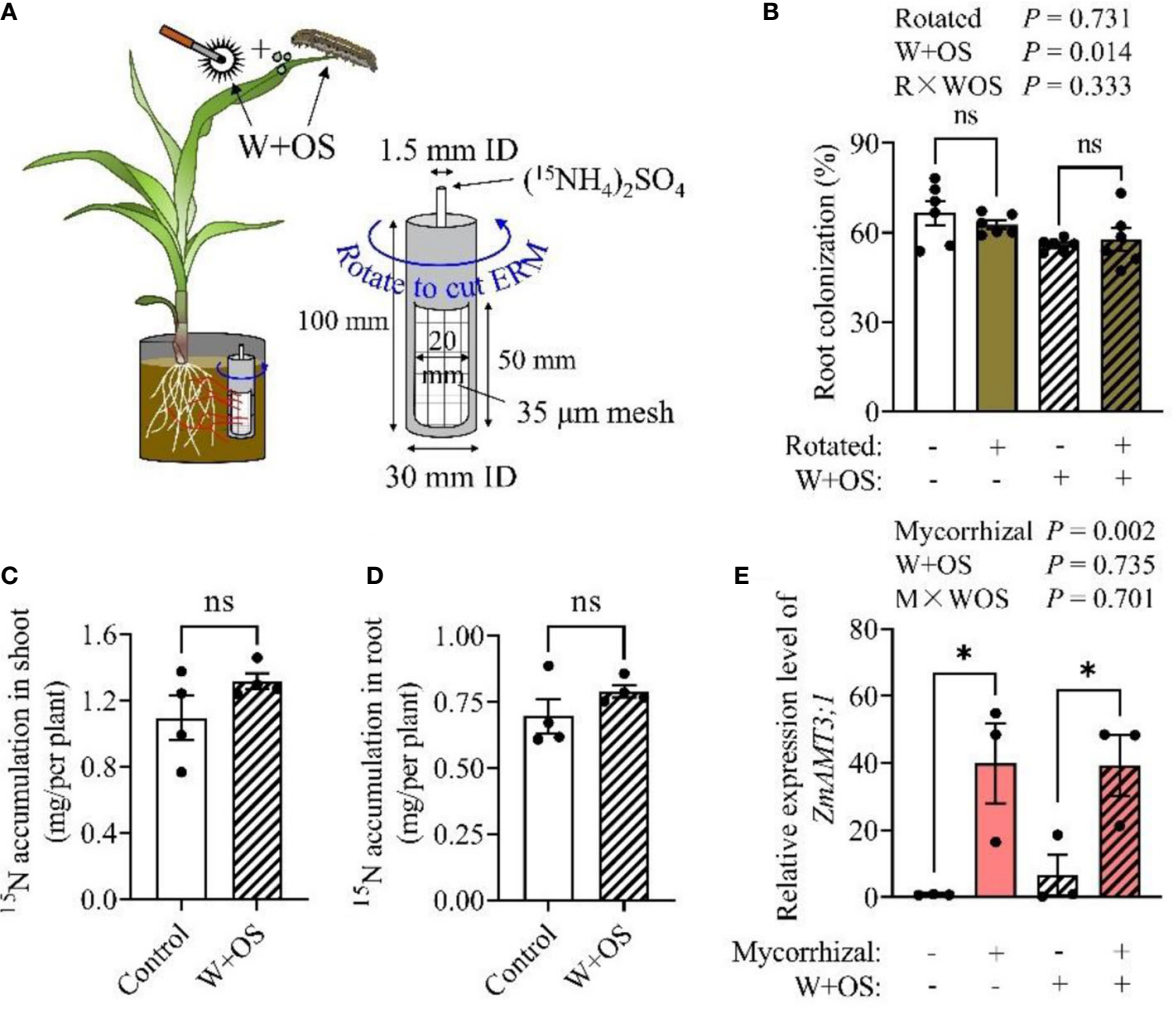
Mycorrhiza-mediated nitrogen uptake from soil by maize plants is independent of aboveground herbivory. Maize plants were inoculated with *Rir* and were cultivated in self-designed boxes, with a PVC core in each box as illustrated in experiment III. Five weeks post *Rir* inoculation, the PVC core was injected with (^15^NH_4_)_2_SO_4_ solution, and was then either rotated daily (for the purpose of severing the mycelium) or nontreated (Intact) for 10 days, during which W+OS treatment was introduced **(A)**. Plants were then harvested to measure the root colonization rate **(B)**, shoot ^15^N **(C)**, root ^15^N **(D)**, and *ZmAMT3;1* (a mycorrhizal inducible nitrogen transporter gene) expression **(E)**. The ^15^N in Intact-’W+OS’ minus the ^15^N in Rotated-’W+OS’ (to counteract the effect of root acquisition of ^15^N) was present as a ‘Control’, and ^15^N in Intact+’W+OS’ minus the ^15^N in Rotated+’W+OS’ (to counteract the effect of herbivory on root acquisition of ^15^N) was present as ‘W+OS’ in **(C)** and **(D)**. NM, non-mycorrhizal roots; AM, arbuscular mycorrhizal roots. For the analysis of root colonization, n = 6; ^15^N accumulation, n = 4; *ZmAMT3;1* expression, n = 3. Data are the means ± SE of biological replicates. Asterisks indicate significant differences (Student’s t-test, *P < 0.05; ns, no significance).

Aboveground herbivorous insects are generally considered to be important external biological sinks for plant carbon and may influence symbiotic relationships and nutrient transfer between plants and AMF by directly competing with AMF for plant carbon resources (Charters et al., 2020). To investigate the effect of aboveground herbivory on maize uptake of N from soil *via* ERM, we used simulated herbivory (Wounding + Oral secretion, W+OS, **Figure S2**, **Figure 3A**) to exclude ^15^N loss due to insect consumption and the differences in *S. frugiperda* feed intake. Our experimental results showed that rotation treatment had no effect on root colonization rate (**Figure 3B**), while W+OS treatment slightly decreased root colonization (12.1% decrease; *P* = 0.014; **Figure 3B**). However, the W+OS treatment did not affect the ammonia uptake of maize plants by the mycorrhizal pathway *via* ERM. There was no significant difference in receiving ^15^N from *Rir* between the control and W+OS treatment in both shoots (**Figure 3C**, **S3A**) and roots (**Figure 3D**, **S3B**). Meanwhile, the expression of the mycorrhizal-induced N transporter gene *ZmAMT3;1* was upregulated during symbiosis (AM), but it was not affected by simulated herbivory (**Figure 3E**, *P* = 0.735), implying that herbivory has no obvious influence on mycorrhiza dependent N uptake. Together, these results together indicated that N uptake by the mycorrhizal pathway from soil in maize is independent of insect herbivory.

### 2.4 N transfer between maize plants mediated by CMNs was inhibited by herbivory

**Figure 4:**
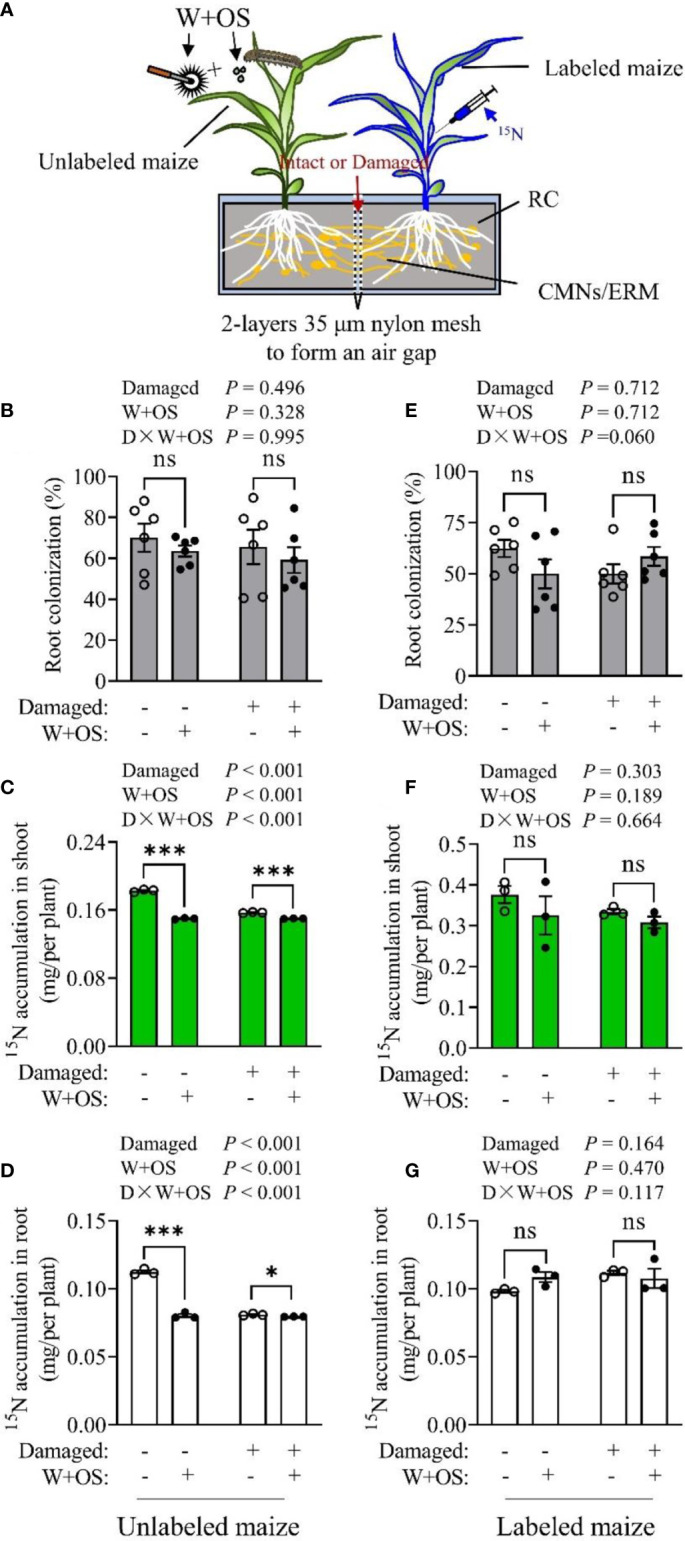
Common mycorrhizal network-mediated N transfer between maize plants was affected by herbivory. Maize plants were inoculated with *Rir* and cultivated in self-designed mesocosms as demonstrated in experiment IV **(A)**, RC, root compartment; CMNs, common mycorrhizal networks; ERM, extraradical mycorrhizal mycelia. The CMNs established between the two maize plants were either damaged daily or intact. Five weeks after *Rir* inoculation the mycorrhizal colonization rate in the roots was measured **(B, E)**. Thereafter, 10 days after ^15^N labeling and W+OS treatment, ^15^N was detected in unlabeled plants **(C, D)** and ^15^N-labeled plants **(F, G)**. Data are the means ± SE of the three (or six) biological replicates. Asterisks indicate significant differences (Student’s t-test, *P < 0.05; ***P < 0.001; ns, no significance).

To examine CMN-mediated N transfer between maize plants, we designed a mesocosm, as illustrated in experiment IV (**Figures S4A, B**), to investigate whether maize plants obtain N from the ^15^N-labeled plants by CMNs (**Figure 4A**). The colonization rates in maize roots were high (**Figures 4B, E**), suggesting that the CMNs between two the maize plants were well established and that *Rir* infection was not affected by CMN damage or herbivory treatment. In ^15^N-labeled plants, the content of ^15^N was higher in the shoot than in the roots, since ^15^N was injected into plant shoots (**Figures 4F, G**; **S5D, E**). In addition, no statistical difference was detected among the different treatments in labeled plant shoots (**Figure 4F**; **S5D**), suggesting that CMN damage and simulated herbivory (W+OS) had no effect on ^15^N transport from the shoot to the roots in labeled maize plants. However, the CMN Intact treatment resulted in increased ^15^N accumulation in unlabeled plant roots (*P<* 0.001, **Figure 4D**; **S5C**) and shoots (*P<* 0.001, **Figure 4C**; **S5B**) compared to the CMN-damaged treatment. These results demonstrated that ^15^N injected in labeled maize shoots could flow to the roots and thus transfer to unlabeled maize plants by CMNs. This further confirmed that the maize plant was able to receive N from its neighboring plant by CMNs as with other plant species described in other previous studies (He et al., 2003; Wang et al., 2016). Interestingly, the same results were observed in W+OS treatment in that the ^15^N accumulation were also reduced both in unlabeled plant roots (*P<* 0.001, **Figure 4D**) and shoots (*P<* 0.001, **Figure 4C**), suggesting that the exportation of ^15^N *via* intact CMNs was restrained due to aboveground W+OS treatment. In addition, the reduced ^15^N accumulation in unlabeled plants caused by W+OS treatment was abolished when the CMNs were damaged. These unlabeled plants with damaged CMNs showed almost the same amount of ^15^N in roots and shoot (**Figures 4C, D**; **S5B, C**). Additionally, the reduction of ^15^N accumulation in unlabeled plants caused by CMN-damage treatment was eliminated by W+OS treatment. These results indicate that aboveground herbivory could block CMN-mediated N transfer from the labeled plant to the unlabeled plant. It is possible that ^15^N could be exudated from the roots of labeled maize plants and therefore, diffuse in soil. Thus, maize plants were each cultivated in a single pot and either ^15^N labeled ((^15^NH_4_)_2_SO_4_ injected in shoot) or nontreated. The soils of the two treatments were then used to detect the exudation of ^15^N from maize in the soil. δ^15^N of labeled maize soil was not significantly different from unlabeled controls (natural ^15^N abundance) (**Figure S6**), implying that there was no ^15^N or only an extremely small amount of ^15^N (derived from (^15^NH_4_)_2_SO_4_) that exuded from the roots of the labeled maize plant during the experimental period. These results suggest that the increased ^15^N accumulation in Intact CMN unlabeled maize plants was obtained through CMNs rather than an extended ERM or root apparatus. Therefore, we confirmed that the CMN-mediated ^15^N transfer between maize plants and the CMN-mediated ^15^N transfer was inhibited by aboveground herbivory.

## 3 Discussion

### 3.1 Multiple mycorrhiza-dependent N uptake pathways in maize

Mycorrhizal symbiosis ubiquitously exists in terrestrial ecosystems (Smith and Smith, 2011; Hazard and Johnson, 2018). The formation and regulation of the symbiotic relationship depend on nutrient reciprocity (Ferlian et al., 2018). The bidirectional exchange of “carbon-mineral nutrients” has been extensively studied (Wipf et al., 2019). Consistent with many previous studies, our results showed that mycorrhizal colonization significantly promoted the biomass accumulation and uptake of mineral nutrients such as N in maize plants (**Figure 1**). N is a crucial nutrient element for plant growth and development in agricultural and natural ecosystems (Crawford and Glass, 1998; Kennedy et al., 2015). The existence of arbuscular mycorrhiza-dependent N uptake pathways in plants has been reported (Govindarajulu et al., 2005; Wang et al., 2020). Therefore, the management of mycorrhiza-dependent N uptake pathways has been considered a feasible strategy for developing sustainable agriculture (Thirkell et al., 2017; Verzeaux et al., 2017), especially for intensive agriculture (Rillig et al., 2019). Our results showed that, except for the root apparatus, mycorrhizal maize plants could also absorb $NH_{4}^{+}$ from soil solution *via* ERM (**Figure 2**).

**Figure 5:**
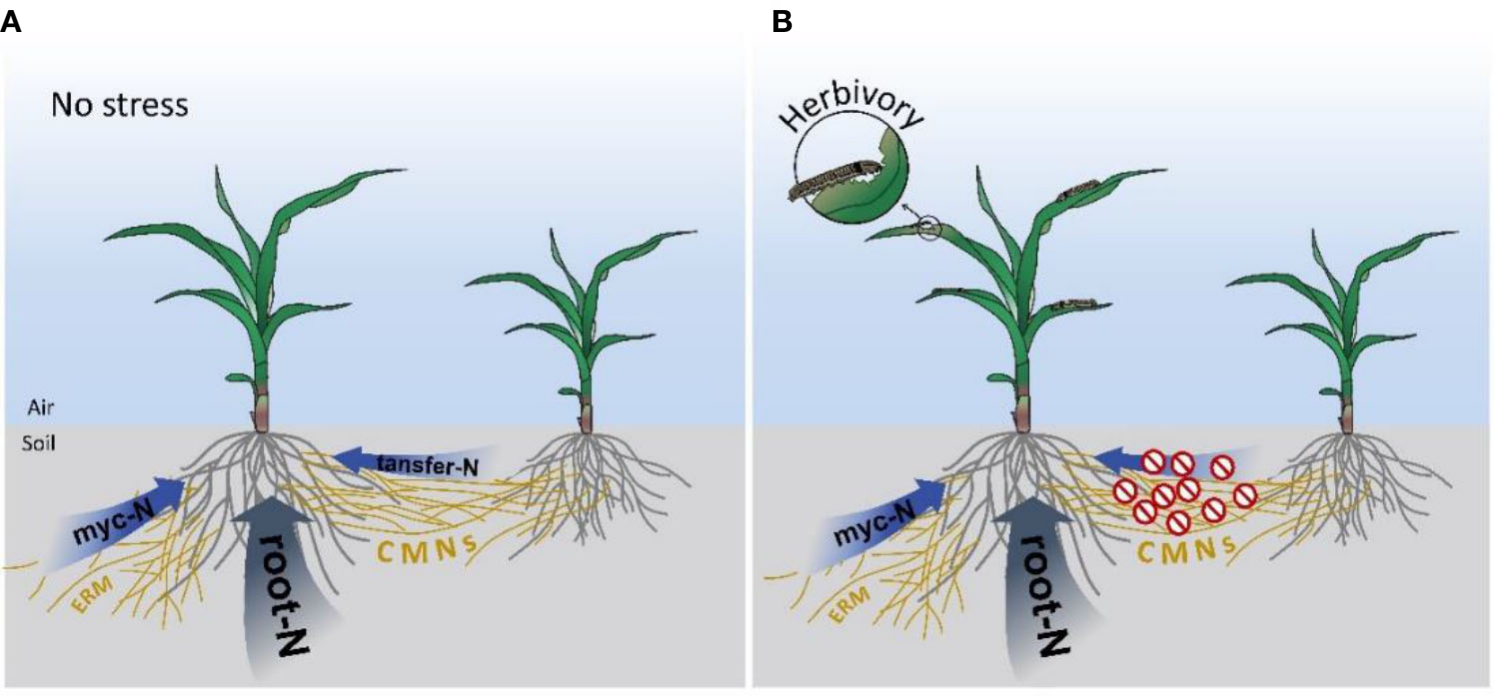
Diagram of pathways for N uptake in maize plants. **(A)** Pathway for maize plant N uptake under no stress. **(B)** Diagram of the pathway for maize plant N uptake under herbivory. Myc-N, N uptake by mycorrhizal pathway from soil; root-N, N uptake by root from soil; transfer-N, N obtained from the CMN-connected neighboring plant; 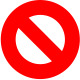, N transferred from a CMN-connected neighboring plant was blocked.

Meanwhile, our experiments confirmed that mycorrhizal maize could acquire N from neighboring plants through CMNs (**Figure 4**). Notably, most of the N delivered by CMNs stayed in the mycorrhizal roots (**Figure S5C**), while only a small amount of labeled ^15^N in shoots was detected (**Figure S5B**), implying that CMNs transferred N from labeled plants might have not been well absorbed and assimilated by maize plants. This may be related to the short time span of our experiments or the need for plant-derived N to complete the complex transformation in the fungus. Nevertheless, the increased accumulation of ^15^N in the shoots of mycorrhizal maize plants suggests that mycorrhizal maize plants could obtain and utilize N from the CMNs connected to neighboring plants. Two recent studies demonstrated that the root-specific and arbuscular mycorrhizal inducible gene, *ZmAMT3;1*, in maize is a high-affinity ammonium transporter that is essential for $NH_{4}^{+}$ transfer to plants *via* the peri-arbuscular membrane, and it contributes about 70% to mycorrhiza-dependent uptake of N (Moulin, 2022.; Hui et al., 2022). Therefore, a large proportion of labeled ^15^ $NH_{4}^{+}$ might be transferred by *ZmAMT3;1* in the mycorrhizal maize roots and thus transported to the shoots. Together, these results suggests that multiple mycorrhiza-dependent N uptake pathways exist in maize plants, by either ERM or CMNs (**Figure 5A**), to improve the utilization of N from different sources.

### 3.2 Aboveground herbivory and N uptake *via* ERM

Plants interact with a variety of other organisms simultaneously in the natural environment, including AMF and aboveground herbivorous insects, and these interactions often affect plant nutrient uptake and utilization (Frew and Price, 2019; Charters et al., 2020). Therefore, the extent to which plants benefit from AMF colonization largely depends on environmental conditions (Pozo et al., 2015). Chewing insects, such as *S. frugiperda*, consume a large proportion of plant biomass (carbon source) (Giri et al., 2006; Nabity et al., 2009). In addition, insect herbivory can influence the carbon source intensity of plants by inducing JA defense signaling and/or altering net photosynthetic rates (Kerchev et al., 2012; Nabity et al., 2013). According to the carbon limitation hypothesis, aboveground herbivory might lead to reduced carbon transfer from host plants to fungi, thereby impairing mycorrhizal colonization (Gehring and Whitham, 1994). In this study, aboveground herbivory slightly inhibited mycorrhizal colonization in maize plants (**Figure 3B**). Other studies have shown that aboveground herbivory can either increase, decrease or have no effect on mycorrhizal colonization (Charters et al., 2020; Frew, 2021; Charters et al., 2022). The well-established symbiotic relationship underlays plant mycorrhiza-dependent mineral nutrient uptake, so that reduced colonization caused by aboveground herbivory might, to some extent, affect N uptake by mycorrhizal maize from soil solutions *via* ERM. However, the results of ^15^N tracer experiments showed that this fraction of N uptake from soil solution by ERM was not affected by aboveground herbivory (**Figures 3C, D**, **S7**). These results are similar to the conclusion by Charters et al. that aboveground aphid infestation does not affect plant mycorrhiza-dependent P uptake (Charters et al., 2020; Charters et al., 2022) but differs from that of Frew (Frew and Price, 2019; Frew, 2021). The variability of these results highlights that host plant–AMF symbiosis and nutrient transfer might be influenced by a range of specific environmental factors, such as host and fungal species, the degree of stress or soil nutrient status (Barto and Rillig, 2010). In addition, the expression of the maize *ZmAMT3;1* gene, which is responsible for $NH_{4}^{+}$ transport in the mycorrhizal pathway, was not affected by aboveground herbivory neither (**Figure 4B**).

### 3.3 A possible mechanism of restriction of CMN-mediated N transfer between maize plants by aboveground herbivory

Numerous studies have demonstrated that N transfer between plants can occur directly through ERM in CMNs (He et al., 2009). Such CMN-mediated N transfer between plants might have practical implications for plant growth, especially in N-deficient ecosystems (Sprent, 2005). Therefore, understanding the CMN-mediated N dynamics and N distribution in agroecosystems is important for exploring crop N utilization. However, it remains unclear whether biotic stress affects CMN-mediated interplant N transfer. Surprisingly, our results showed that aboveground herbivory blocked the CMN-mediated transfer of ^15^N from the labeled neighboring plants (**Figure 4**). Similarly, N accumulation promoted by CMNs was also inhibited by aboveground herbivory (**Figure S8**). This implies that aboveground herbivory not only affects the CMN-dependent N uptake of host maize, but also regulates maize–plant interactions through CMNs. At present, the molecular mechanism by which aboveground herbivory affects CMN-mediated mineral nutrients transfer is still unknown. However, several studies have shown that CMN-mediate interplant defense–signaling transfer. As Song et al. reported that CMNs could mediate disease defense signaling between tomato plants (Song et al., 2010), CMNs have served as a “pipeline” for defense signaling transfer in various mycorrhizal plants, including *Vicia faba* L (Babikova et al., 2013; Babikova et al., 2013; Cabral et al., 2019)., citrus (*Poncirus trifoliata* L.) (Zhang et al., 2019), and potato (*Solanum tuberosum* L.) (Alaux et al., 2020). A recent study on tobacco (*Nicotiana attenuata*) showed that the ERMs of CMNs expanded the scale of plant defenses and might act as a channel for the transmission of defense signals and terpenoids that help neighboring plants build better defense responses (Song et al., 2019). These findings highlight the complexity of studying the ecological functions of CMNs.

Resource relocation is an important strategy for plant tolerance to insect herbivory (Züst and Agrawal, 2017). Reallocation of key nutrients such as N is vital for plant tolerance to insect herbivory. Upon aboveground herbivory plants may shut down the exchange from neighboring plants *via* CMNs to enhance plant tolerance to insect herbivory although the detailed mechanism is still unknown. Therefore, we speculate that when mycorrhizal maize plants are at risk of herbivory, they use widespread underground CMNs to transmit ‘danger signals’ to neighboring plants, and the ‘warned’ neighboring plants limit CMN-mediated interplant N transfer for better survival under the coming herbivory (**Figure 5B**).

N is a key nutrient element in both natural and agricultural ecosystems. Mycorrhiza-dependent N acquisition and CMN-mediated N transfer between neighboring plants have important ecological implications. Although the molecular basis for how nutrient transfer and stress signaling occur is lacking, it is clear that CMN-mediated distribution and transmission of such nutrients, chemicals, and defense signaling has broad implications for ecosystems, pest management, and sustainable agriculture (Bücking et al., 2016; Oelmüller, 2019).

## 4 Materials and methods

### 4.1 Plant growth

Maize seeds (Zhengdan 958, purchased from Shandong Luyan Agricultural Seed Co., Ltd., China) were sterilized by 2% NaClO for 10 min and then placed in a seedling substrate (German Dahan peat soil, type: 413, particle size: 0-6 mm) for germination. After 7 d, consistent healthy seedlings were selected for transplantation for subsequent experiments. The maize plants were cultivated in an artificial intelligence greenhouse (14 h/10 h, light/dark) with a day and night temperature regime of 30°C (16 h) and 25°C (8 h). The relative humidity of the greenhouse was 70%. The maize plants were properly watered with low-phosphorus Hoagland nutrient solution.

### 4.2 Arbuscular mycorrhizal fungi colonization

The original strain of *Rhizophagus irregularis* (*Rir*, BGC GD01C) was obtained from the Institute of Plant Nutrition and Resources, Beijing Academy of Agriculture and Forestry. Maize plants grown in sands were used for reproduction of *Rir* stain. For mycorrhizal inoculation, the inocula (the mixture of sands, spores, mycelia and root segments) were mixed with high-temperature sterilized sands in a ratio of 1:6, and then were put into plastic pots in which the maize plants were planted.

### 4.3 Insect culture

The original insect population of *Spodoptera frugiperda* used in the experiments was obtained from the laboratory of Professor Lin Jintian, Zhongkai University of Agriculture and Engineering, and was raised and bred for more than 10 generations on artificial diets without exposure to any pesticides. The adults were raised in 10% honey water. The rearing chamber was kept under constant environmental conditions (25 ± 2°C, 60% RH, L:D = 16:8 h).

### 4.4 Experiment I: effect of mycorrhizae on host maize growth and plant nutrition uptake

A box was separated into two equal-sized compartments by two layers of 30–35 μm nylon mesh (which allowed passage of AMF mycelia but not roots) with an interval of 1 cm (**Figure 1A**). Maize plants grown in the left compartment were either inoculated with *Rir* (AM) or with high-temperature sterilized *Rir* (NM; 121°C for 1 h twice). For AM treatment, the mycelium could extend into the right compartment to absorb the substances *via* the mycorrhizal pathway. Five weeks post inoculation, maize plants were harvested for the analysis of dry weight and nutrient content.

### 4.5 Experiment II: N uptake of mycorrhizal maize *via* ERM

At the time of planting (all maize plants were inoculated with *Rir*), PVC cores, each with a stainless-steel tube in the center (made according to Charters et al. (Charters et al., 2020)), were inserted into the pot (15 cm × Φ13 cm), with one PVC core per pot (**Figure 2A**). Five weeks post-planting, the pot was filled with AMF ERM, and then a dose of ^15^N (2.2 mg) was introduced into the central tubes in an aqueous solution. The ^15^N solution was added every two days for all PVC cores. Half of the PVC cores were rotated every day to sever the connection of the ERM inside and outside the PVC core and set as the ‘Rotated’ treatment; the other half of the PVC cores were not rotated and set as the ‘Intact’ treatment. Ten days later, maize plants were harvested to determine ^15^N uptake.

### 4.6 Experiment III: herbivory effect on N uptake of mycorrhizal maize *via* ERM

The design of this experiment is shown in **Figure 3A**, and the maize leaves were treated with simulated herbivory. Maize plant cultivation, ^15^N labeling, and PVC core rotation treatment were conducted as described in experiment II. To minimize consumption differences and ^15^N losses due to insect chewing, insect herbivory was simulated by mechanical wounding + oral secretions (W+OS) of *S. frugiperda* larvae. Oral secretions were collected as described in **Figure S2**. ^15^N injection and PVC core rotation were carried out every two days, five times in total) (**Figure 3A**). Thus, four treatment groups in this part of experiment, included: Rotated-’W+OS’, Intact-’W+OS’, Rotated+’W+OS’, and Intact+’W+OS’. Among the four treatment groups, Rotated-’W+OS’ and Rotated+’W+OS’ were used to exclude experimental errors caused by direct absorption of ^15^N by plant roots due to soil solution flow or soil capillarity. Therefore, the value of ^15^N in Intact-’W+OS’minus the value of ^15^N in Rotated-’W+OS’ was present as the ‘control’ in the final result, and the value of ^15^N in Intact+’W+OS’ minus the value of ^15^N in Rotated+’W+OS’ was present as ‘W+OS’ in the final result. Ten days post-treatment, the plants were harvested for biomass and ^15^N content analyses.

### 4.7 Experiment IV: N transfer between maize plants mediated *via* CMNs

The mesocosm design for this experiment was based on the classical compartmental partition method (Song et al., 2010). The mesocosm design schematic diagram is shown in **Figure S4A**. The mesocosm frame was made of a box (length: width: height = 30: 18: 15 cm), and the box was separated into two equal-sized compartments by two layers of 30–35 μm nylon mesh (which excluded root contact between plants but allowed the passage of ERM) with an interval of 1 cm. The narrow interval space between the two layers of nylon mesh was called the mycelia compartment (MC). Both the left and right compartments were planted with maize plants, and the compartment with plant roots was named the root compartment (RC). Maize plants grown in the left RC were inoculated with *Rir*, while maize plants grown in the right RC were non-inoculated. The mycelium of the mycorrhizal maize roots in the left RC could pass through the nylon mesh and thus reinfect the non-inoculated maize roots to form CMNs. The purpose of setting double-layer nylon mesh was mainly to increase the distance of the root-free zone between two maize plant roots, which could prevent the direct transfer of nutrients, water or root exudates between the two root compartments. Five weeks post *Rir* inoculation, the CMNs between two maize plants were well established (by detecting the mycorrhizal rate of maize roots in the right RC). ^15^N labeling was conducted every two days by directly injecting the ^15^N solution into the stem of the maize plant in the right RC (labeled plant). For CMN damage treatment, a steel ruler was inserted into the air gap between two-layer nylon mesh to cut the mycelium back and forth every day. Insect herbivory proceeded on unlabeled plants was simulated by mechanical wounding and oral secretions (W+OS) of *S. frugiperda* larvae (**Figure S2B**). Ten days later, the plants were harvested to measure for dry weight and analyze the ^15^N content.

### 4.8 Oral secretion collection and simulated herbivory

Oral secretions (OS) were collected from the 5th instar *S. frugiperda* larvae fed on maize seedlings (**Figure S2A**). The larval mouth was touched with a 0.1-10 µL pipette tip to stimulate the larvae to spit out oral secretions (**Figure S2B**). The oral secretions of the larvae were quickly pipetted and collected in pre-chilled 1.5 mL EP tubes (**Figure S2C**) and centrifuged at 12,000 rpm for 10 min at 4°C to remove food debris. The oral secretions were finally diluted 5-fold with ddH_2_O before use (**Figure S2D**). For wounding and OS-elicitation treatment (W+OS), maize seedlings with the same growth phenotype were selected for the W+OS treatment. A barbed roller dipped with the OS was used to pierce each leaf of the maize plants to imitate the herbivorous insect (**Figure S2E**). The number of piercing holes was determined according to the size of the leaf, generally 50–100 holes/leaf (**Figure S2F**). The treatment was repeated every three days, three times during the test period, and the samples were collected after 10 days of treatment.

### 4.9 ^15^N labeling

Ammonium sulfate-^15^N_2_, ((^15^NH_4_)_2_SO_4_; CAS: 43086-58-4; abundance: 99 atom %, chemical purity: ≥ 98.5%; Shanghai Stable Isotope Engineering Technology Research Center) was used for ^15^N labeling. ^15^N labeling in maize plants was performed after the plants had grown to the V6 or 6-leaf stage to the V10 or 10-leaf stage. In the experiment, maize plants were labeled with ^15^N by the stem injection method. (^15^NH_4_)_2_SO_4_ (0.118 g) was dissolved in 10 mL ddH_2_O to prepare ^15^N labeling solution. Using a micro-syringe, the ^15^N labeling solution was injected into the main stem of maize plants. Each plant received 10 µL ^15^N labeling solution every two days, continuously five times. For the unlabeled plant in the same apparatus, an equal amount of unlabeled nitrogen was injected.

The stable isotopes of ^15^N were analyzed and determined using a trace gas pre-concentration system-stable isotope ratio mass spectrometer (UK, model: Isoprime100). The determination process was completed at the Forest Ecology-Stable Isotope Research Center, School of Forestry, Fujian Agriculture and Forestry University. ^15^N abundance and accumulation were as calculated as follows:


$$(A){\;}^{15}N\;abundance(atom\%)=[(Number\;of{\;}^{15}N\;atoms)/(Number\;of{\;}^{14}N{+}^{15}N\;atoms)]\times 100$$

$$(B)\;\delta^{15}N(‰)=[{(}^{15}N{/}^{14}N_{sample}{-}^{15}N{/}^{14}N_{s\tan dard}){/}^{15}N{/}^{14}N_{s\tan dard}]\times 1000$$

$$(C){\;}^{15}N_{accumulation}=atom{\%}^{15}N_{sample}\times total\;content\;N_{sample}$$

### 4.10 Plant harvest

Maize plant shoots and roots of experiment I−IV were separately harvested. After washing, the roots were dried on a paper towel, and one half were immediately frozen in liquid nitrogen RNA extraction. The rest of roots and the shoots were oven-dried to constant weight at 60°C. Totally twelve plants were harvested in each treatment group. For the determination of the mycorrhizal infection rate from two independent root samples were mixed into one biological replicate resulting in six biological replicates. For the measurements of ^15^N accumulation, four independent plant samples were combined into one biological sample resulting in three biological replicates.

### 4.11 Root staining and mycorrhizal colonization assessment

The staining method was slightly modified from Song et al. (Song et al., 2019). The fresh maize root segments were placed in a centrifuge tube containing 10% KOH and heated at 96°C for 5 min. The roots were washed with sterile water three times to remove the remaining KOH and placed in a centrifuge tube containing 2% HCl at 96°C for 5 min. After washing several times with 0.2 M PBS (pH = 7.4), the roots were re-immersed in PBS and placed at room temperature for 3 h. The roots were then transferred to PBS containing 5 µg/mL WGA-488 (wheat germ agglutinin-Alexa fluor 488 conjugate, Invitrogen, W11261) and stained overnight at 4°C. The stained root segments were washed several times with PBS and then placed in 0.2 M PBS containing 10 µg/mL PI for 1 h at room temperature, followed by washing with PBS. Stained root segments were observed under a Nikon Eclipse Ti2 inverted fluorescence microscope (Shanghai Nikon Instruments Co., Ltd., China), with an excitation wavelength of 488 nm and an emission wavelength of 507 nm, and photographed with a monochrome microscope camera (Nikon DS-Ri2). The mycorrhizal colonization rate was calculated as described by Biermann et al. (Biermann and Linderman, 1981).

### 4.12 Measurement of plant height and biomass

Forty days post *Rir* inoculation, the mycorrhizal mycelial network was established. The plant vertical distance from the stem base of the plant to the highest point in the natural state of the plant was measured as the natural height of maize plants. The fresh plants were harvested, and then placed into an oven with 60°C constant temperature to constant weight.

### 4.13 Determination of plant phosphorus content

The phosphorus content was determined using Bao’s method (Bao, 2000), with slight modifications. Maize plants were harvested and cut into small pieces (0-2 mm) with scissors, and then were ground into powder. The powder (0.100 g) was placed into a digestion tube (Denmark, FOSS), and 5 mL of HNO_3_ were added to digest the sample at room temperature for 12 h, followed by further digestion at 180°C for 30 min on the digestion furnace. The digested solution was made up to 50 mL, shaken, mixed, filtered, and centrifuged at 4000 rpm for 5 min; 10 μL of the supernatant was added to the molybdenum antimony anti-staining agent and incubated at 42°C for 20 min. The absorbance was detected at OD_820nm_.

### 4.14 Determination of total nitrogen content

The Kjeldahl method, with slight modifications, was used to determine the nitrogen content (Bao, 2000). For H_2_SO_4_-H_2_O_2_ digestion, 0.100 g of the plant powder sample was placed in a digestive tube (provided with the FOSS Kjeldahl nitrogen analyzer), and 2 g catalyst (Cu_2_SO_4_: KCl=9:1, m/m) and 5 mL of concentrated sulfuric acid were added. The sample was then digested at 400°C for about 60 min. During digestion, drops of 300 g/L H_2_O_2_ were added (after cooling the digestion tube) until the digestion liquid became colorless or clear. The total nitrogen content in the digested plant samples was determined with a FOSS automatic Kjeldahl nitrogen analyzer (Denmark, model: Kjeltec 8400). The detailed procedure was performed according to the instrument manual for the operation process.

### 4.15 Quantitative real-time PCR analysis

Procedures used for RNA extraction and reverse transcription of plant samples were carried out as previously described (Lin et al., 2019), with slight modifications. Total RNA was extracted from ~0.1 g flash-frozen, powdered root samples using the Eastep^®^ Super Total RNA Extraction kit (Promega Biotech *Co., Ltd*., China) according to the manufacturer’s instructions. Total RNA was treated with RNase-Free DNaseI (TIANGEN Biotech *Co., Ltd*., China), and 1 μg of total RNA was pipetted for cDNA synthesis using the GoScript Reverse Transcription System (Promega Biotech *Co., Ltd*., China). Real-time PCR was performed using the MonAmp ChemoHS qPCR Mix (High Rox) Kit (Monad Biotech *Co., Ltd*., China). Reaction conditions for thermal cycling were 95°C for 5 min, followed by 40 cycles of 95°C for 10 s, 55–65°C for 10 s, and 72°C for 30 s. Fluorescence data were collected during the cycle at 72°C. The gene expression level was normalized using the maize housekeeping gene *GAPDH* and the 2^-ΔΔCT^ method. The gene-specific primers used in this research are listed in **Table S1**. Biological triplicates with technical duplicates were performed.

### 4.16 Statistical analysis

Data were processed and plotted using Microsoft Excel 2013 and GraphPad Prism 9 software, and significance was tested using SPSS 19. All experiments were conducted using a completely randomized experimental design. Data were checked for normality (*P* > 0.05) using the Shapiro-Wilk normality test and Levene’s test for homogeneity of variance (*P* > 0.05) prior to all statistical analyses. On the premise of satisfying the assumption of normality and homogeneity of variance, a Student’s t-test or analysis of variance (ANOVA) (Tukey’s *post hoc* test, *P*< 0.05) were used to compare the differences between two or more treatments. When the assumptions of normality and variance homogeneity were not met, the Mann-Whitney U test or the Kruskal–Wallis test were used to analyze the data of two or more groups, and then Fisher’s least significant difference (LSD) and the Holm correction adjusted for P values were used to compare significant differences.

## Data availability statement

The original contributions presented in the study are included in the article/**Supplementary Material**. Further inquiries can be directed to the corresponding authors.

## Author contributions

YL, YS, DC and RZ conceived and designed the experiments, and wrote the manuscript. CH, YL, LT, YZ, YD, MY and QL carried out the experiments. YS, CH., YL. and RZ analyzed the data and contributed to the discussion and revision. All authors contributed to the article and approved the submitted version.

## References

[B1] Alaux P.-L. Naveau F. Declerck S. Cranenbrouck S. (2020). Common mycorrhizal network induced JA/ET genes expression in healthy potato plants connected to potato plants infected by phytophthora infestans. Front. Plant Sci. 11. doi: 10.3389/fpls.2020.00602 PMC7261899 32523589

[B2] Almario J. Fabiańska I. Saridis G. Bucher M. (2022). Unearthing the plant–microbe quid pro quo in root associations with beneficial fungi. New Phytol. 234 (6), 1967–1976. doi: 10.1111/nph.18061 35239199

[B3] An J. Zeng T. Ji C. de Graaf S. Zheng Z. Xiao T. T.. (2019). A medicago truncatula SWEET transporter implicated in arbuscule maintenance during arbuscular mycorrhizal symbiosis. New Phytol. 224 (1), 396–408. doi: 10.1111/nph.15975 31148173

[B4] Babikova Z. Gilbert L. Bruce T. J. Birkett M. Caulfield J. C. Woodcock C.. (2013). Underground signals carried through common mycelial networks warn neighbouring plants of aphid attack. Ecol. Lett. 16 (7), 835–843. doi: 10.1111/ele.12115 23656527

[B5] Babikova Z. Johnson D. Bruce T. Pickett J. Gilbert L. (2013). How rapid is aphid-induced signal transfer between plants via common mycelial networks? Commun. Integr. Biol. 6 (6), 835–843. doi: 10.4161/cib.25904 PMC3917958 24563703

[B6] Bao S. (2000). Soil agrochemical analysis (Beijing: China Agricultural Press), 30.

[B7] Barto E. K. Rillig M. C. (2010). Does herbivory really suppress mycorrhiza? a meta-analysis. J. Ecol. 98 (4), 745–753. doi: 10.1111/j.1365-2745.2010.01658.x

[B8] Biermann B. Linderman R. (1981). Quantifying vesicular-arbuscular mycorrhizae: a proposed method towards standardization. New Phytol. 87 (1), 63–67. doi: 10.1111/j.1469-8137.1981.tb01690.x

[B9] Boldt K. Pörs Y. Haupt B. Bitterlich M. Kühn C. Grimm B.. (2011). Photochemical processes, carbon assimilation and RNA accumulation of sucrose transporter genes in tomato arbuscular mycorrhiza. J. Plant Physiol. 168 (11), 1256–1263. doi: 10.1016/j.jplph.2011.01.026 21489650

[B10] Bücking H. Mensah J. A. Fellbaum C. R. (2016). Common mycorrhizal networks and their effect on the bargaining power of the fungal partner in the arbuscular mycorrhizal symbiosis. Commun. Integr. Biol. 9 (1), e1107684. doi: 10.1080/19420889.2015.1107684 27066184 PMC4802747

[B11] Cabral C. Wollenweber B. António C. Ravnskov S. (2019). Activity in the arbuscular mycorrhizal hyphosphere warning neighbouring plants. Front. Plant Sci. 10. doi: 10.3389/fpls.2019.00511 PMC6482268 31057597

[B12] Charters M. D. Durant E. K. Sait S. M. Field K. J. (2022). Impacts of aphid herbivory on mycorrhizal growth responses across three cultivars of wheat. Plants. People. Planet 4 (6), 655–666. doi: 10.1002/ppp3.10302

[B13] Charters M. D. Sait S. M. Field K. J. (2020). Aphid herbivory drives asymmetry in carbon for nutrient exchange between plants and an arbuscular mycorrhizal fungus. Curr. Biol. 30 (10), 1801–1808. e5. doi: 10.1016/j.cub.2020.02.087 32275877 PMC7237887

[B14] Choi J. Summers W. Paszkowski U. (2018). Mechanisms underlying establishment of arbuscular mycorrhizal symbioses. Annu. Rev. Phytopathol. 56, 135–160. doi: 10.1146/annurev-phyto-080516-035521 29856935

[B15] Cliquet J. B. Murray P. J. Boucaud J. (1997). Effect of the arbuscular mycorrhizal fungus glomus fasciculatum on the uptake of amino nitrogen by lolium perenne. New Phytol. 137 (2), 345–349. doi: 10.1046/j.1469-8137.1997.00810.x 33863187

[B16] Crawford N. M. Glass A. D. (1998). Molecular and physiological aspects of nitrate uptake in plants. Trends Plant Sci. 3 (10), 389–395. doi: 10.1016/S1360-1385(98)01311-9

[B17] Etesami H. Adl S. M. (2020). Plant growth-promoting rhizobacteria (PGPR) and their action mechanisms in availability of nutrients to plants. In: Kumar M. Kumar V. Prasad R. editors, Phyto-Microbiome. Stress Regul. Environmental and Microbial Biotechnology. (Singapore: Springer). 147–203. doi: 10.1007/978-981-15-2576-6_9

[B18] Ferlian O. Biere A. Bonfante P. Buscot F. Eisenhauer N. Fernandez I.. (2018). Growing research networks on mycorrhizae for mutual benefits. Trends Plant Sci. 23 (11), 975–984. doi: 10.1016/j.tplants.2018.08.008 30241736 PMC6370000

[B19] Frew A. (2021). Aboveground herbivory suppresses the arbuscular mycorrhizal symbiosis, reducing plant phosphorus uptake. Appl. Soil Ecol. 168, 104133. doi: 10.1016/j.apsoil.2021.104133

[B20] Frew A. Price J. N. (2019). Mycorrhizal-mediated plant–herbivore interactions in a high CO_2_ world. Funct. Ecol. 33 (8), 1376–1385. doi: 10.1111/1365-2435.13347

[B21] Frey B. Schüepp H. (1993). Acquisition of nitrogen by external hyphae of arbuscular mycorrhizal fungi associated with zea mays l. New Phytol. 124 (2), 221–230. doi: 10.1111/j.1469-8137.1993.tb03811.x 33874357

[B22] Gehring C. A. Whitham T. G. (1994). Interactions between aboveground herbivores and the mycorrhizal mutualists of plants. Trends Ecol. Evol. 9 (7), 251–255. doi: 10.1016/0169-5347(94)90290-9 21236843

[B23] Ge L. Sun S. Chen A. Kapulnik Y. Xu G. (2008). Tomato sugar transporter genes associated with mycorrhiza and phosphate. Plant Growth Regul. 55 (2), 115–123. doi: 10.1007/s10725-008-9266-7

[B24] Giri A. P. Wunsche H. Mitra S. Zavala J. A. Muck A. Svatosš A.. (2006). Molecular interactions between the specialist herbivore manduca sexta (Lepidoptera, sphingidae) and its natural host *Nicotiana attenuata.* VII. changes in the plant's proteome. Plant Physiol. 142 (4), 1621–1641. doi: 10.1104/pp.106.088781 17028148 PMC1676057

[B25] Govindarajulu M. Pfeffer P. E. Jin H. Abubaker J. Douds D. D. Allen J. W.. (2005). Nitrogen transfer in the arbuscular mycorrhizal symbiosis. Nature 435 (7043), 819–823. doi: 10.1038/nature03610 15944705

[B26] Hazard C. Johnson D. (2018). Does genotypic and species diversity of mycorrhizal plants and fungi affect ecosystem function? New Phytol. 220 (4), 1122–1128. doi: 10.1111/nph.15010 29393517

[B27] He X.-H. Critchley C. Bledsoe C. (2003). Nitrogen transfer within and between plants through common mycorrhizal networks (CMNs). Crit. Rev. Plant Sci. 22 (6), 531–567. doi: 10.1080/713608315

[B28] He X. Critchley C. Ng H. Bledsoe C. Reciprocal N. (2004). (^15^ $NH_{4}^{+}$ or ^15^ $NO_{3}^{-}$ ) transfer between nonN_2_-fixing eucalyptus maculata and N_2_-fixing *Casuarina cunninghamiana* linked by the ectomycorrhizal fungus *Pisolithus* sp. New Phytol. 163 (3), 629–640. doi: 10.1111/j.1469-8137.2004.01137.x 33873747

[B29] He X. Xu M. Qiu G. Y. Zhou J. (2009). Use of ^15^N stable isotope to quantify nitrogen transfer between mycorrhizal plants. J. Plant Ecol. 2 (3), 107–118. doi: 10.1038/s41598-021-90436-8

[B30] Hodge A. Storer K. (2015). Arbuscular mycorrhiza and nitrogen: Implications for individual plants through to ecosystems. Plant Soil 386 (1), 1–19. doi: 10.1007/s11104-014-2162-1

[B31] Hui J. An X. Li Z. Neuhäuser B. Ludewig U. Wu X.. (2022). The mycorrhiza-specific ammonium transporter *ZmAMT3; 1* mediates mycorrhiza-dependent nitrogen uptake in maize roots. Plant Cell 34 (10), 4066–4087. doi: 10.1093/plcell/koac225 35880836 PMC9516061

[B32] Jaiswal S. K. Mohammed M. Ibny F. Y. Dakora F. D. (2021). Rhizobia as a source of plant growth-promoting molecules: Potential applications and possible operational mechanisms. Front. Sustain. Food Syst. 4. doi: 10.3389/fsufs.2020.619676

[B33] Jiang Y. Wang W. Xie Q. Liu N. Liu L. Wang D.. (2017). Plants transfer lipids to sustain colonization by mutualistic mycorrhizal and parasitic fungi. Science 356 (6343), 1172–1175. doi: 10.1126/science.aam9970 28596307

[B34] Johansen A. Jensen E. (1996). Transfer of n and p from intact or decomposing roots of pea to barley interconnected by an arbuscular mycorrhizal fungus. Soil Biol. Biochem. 28 (1), 73–81. doi: 10.1016/0038-0717(95)00117-4

[B35] Kennedy P. Walker J. Bogar L. Horton T. (2015). Interspecific mycorrhizal networks and non-networking hosts: Exploring the ecology of the host genus Alnus. (Springer Netherlands Dordrecht), 227–254. Available at: https://www.springer.com/gp/book/9789401773942.

[B36] Kerchev P. I. Fenton B. Foyer C. H. Hancock R. D. (2012). Plant responses to insect herbivory: interactions between photosynthesis, reactive oxygen species and hormonal signalling pathways. Plant. Cell Environ. 35 (2), 441–453. doi: 10.1111/j.1365-3040.2011.02399.x 21752032

[B37] Kobae Y. Tamura Y. Takai S. Banba M. Hata S. (2010). Localized expression of arbuscular mycorrhiza-inducible ammonium transporters in soybean. Plant Cell Physiol. 51 (9), 1411–1415. doi: 10.1093/pcp/pcq099 20627949

[B38] Koegel S. Ait Lahmidi N. Arnould C. Chatagnier O. Walder F. Ineichen K.. (2013). The family of ammonium transporters (AMT) in s orghum bicolor: Two AMT members are induced locally, but not systemically in roots colonized by arbuscular mycorrhizal fungi. New Phytol. 198 (3), 853–865. doi: 10.1111/nph.12199 23461653

[B39] Lin Y. Sun Z. Li Z. Xue R. Cui W. Sun S.. (2019). Deficiency in silicon transporter Lsi1 compromises inducibility of anti-herbivore defense in rice plants. Front. Plant Sci. 10. doi: 10.3389/fpls.2019.00652 PMC6543919 31178878

[B40] Ma X. Geng Q. Zhang H. Bian C. Chen H. Y. Jiang D.. (2021). Global negative effects of nutrient enrichment on arbuscular mycorrhizal fungi, plant diversity and ecosystem multifunctionality. New Phytol. 229 (5), 2957–2969. doi: 10.1111/nph.17077 33188641

[B41] Merckx V. S. Gomes S. I. (2020). Symbiosis: Herbivory alters mycorrhizal nutrient exchange. Curr. Biol. 30 (10), R437–R439. doi: 10.1016/j.cub.2020.04.016 32428473

[B42] Moulin S. (2022). Get connected to the fungal network for improved transfer of nitrogen: the role of ZmAMT3; 1 in ammonium transport in maize-arbuscular mycorrhizal symbiosis. Plant Cell 34, 3509–3511. doi: 10.1093/plcell/koac221 35929506 PMC9516037

[B43] Nabity P. D. Zavala J. A. DeLucia E. H. (2009). Indirect suppression of photosynthesis on individual leaves by arthropod herbivory. Ann. Bot. 103 (4), 655–663. doi: 10.1093/aob/mcn127 18660492 PMC2707346

[B44] Nabity P. D. Zavala J. A. DeLucia E. H. (2013). Herbivore induction of jasmonic acid and chemical defences reduce photosynthesis in nicotiana attenuata. J. Exp. Bot. 64 (2), 685–694. doi: 10.1093/jxb/ers364 23264519 PMC3542056

[B45] Oelmüller R. (2019). Interplant communication via hyphal networks. Plant Physiol. Rep. 24 (4), 463–473. doi: 10.1111/nph.13115

[B46] Oerke E.-C. (2006). Crop losses to pests. J. Agric. Sci. 144 (1), 31–43. doi: 10.1017/S0021859605005708

[B47] Paredes-Sánchez F. A. Rivera G. Bocanegra-García V. Martínez-Padrón H. Y. Berrones-Morales M. Niño-García N.. (2021). Advances in control strategies against spodoptera frugiperda. A review. Molecules 26 (18), 5587. doi: 10.3390/molecules26185587 34577058 PMC8471127

[B48] Pérez-Tienda J. Testillano P. S. Balestrini R. Fiorilli V. Azcón-Aguilar C. Ferrol N. (2011). *GintAMT2*, a new member of the ammonium transporter family in the arbuscular mycorrhizal fungus glomus intraradices. Fungal Genet. Biol. 48 (11), 1044–1055. doi: 10.1016/j.fgb.2011.08.003 21907817

[B49] Pozo M. J. López-Ráez J. A. Azcón-Aguilar C. García-Garrido J. M. (2015). Phytohormones as integrators of environmental signals in the regulation of mycorrhizal symbioses. New Phytol. 205 (4), 1431–1436. doi: 10.1111/nph.13252 25580981

[B50] Prasanna B. M. Bruce A. Beyene Y. Makumbi D. Gowda M. Asim M.. (2022). Host plant resistance for fall armyworm management in maize: Relevance, status and prospects in Africa and Asia. Theor. Appl. Genet. 135, 1–20. doi: 10.1007/s00122-022-04073-4 PMC9729323 35320376

[B51] Rillig M. C. Aguilar-Trigueros C. A. Camenzind T. Cavagnaro T. R. Degrune F. Hohmann P.. (2019). Why farmers should manage the arbuscular mycorrhizal symbiosis. New Phytol. 222 (3), 1171–1175. doi: 10.1111/nph.15602 30657593

[B52] Ruzicka D. R. Hausmann N. T. Barrios-Masias F. H. Jackson L. E. Schachtman D. P. (2012). Transcriptomic and metabolic responses of mycorrhizal roots to nitrogen patches under field conditions. Plant Soil 350 (1), 145–162. doi: 10.1007/s11104-011-0890-z

[B53] Smith S. E. Smith F. A. (2011). Roles of arbuscular mycorrhizas in plant nutrition and growth: New paradigms from cellular to ecosystem scales. Annu. Rev. Plant Biol. 62, 227–250. doi: 10.1146/annurev-arplant-042110-103846 21391813

[B54] Smith S. E. Smith F. A. Jakobsen I. (2003). Mycorrhizal fungi can dominate phosphate supply to plants irrespective of growth responses. Plant Physiol. 133 (1), 16–20. doi: 10.1104/pp.103.024380 12970469 PMC1540331

[B55] Smith S. E. Smith F. A. Jakobsen I. (2004). Functional diversity in arbuscular mycorrhizal (AM) symbioses: the contribution of the mycorrhizal p uptake pathway is not correlated with mycorrhizal responses in growth or total p uptake. New Phytol. 162 (2), 511–524. doi: 10.1111/j.1469-8137.2004.01039.x

[B56] Song Y. Wang M. Zeng R. Groten K. Baldwin I. T. (2019). Priming and filtering of antiherbivore defences among nicotiana attenuata plants connected by mycorrhizal networks. Plant. Cell Environ. 42 (11), 2945–2961. doi: 10.1111/pce.13626 31348534

[B57] Song Y. Y. Zeng R. S. Xu J. F. Li J. Shen X. Yihdego W. G. (2010). Interplant communication of tomato plants through underground common mycorrhizal networks. PloS One 5 (10), e13324. doi: 10.1371/journal.pone.0013324 20967206 PMC2954164

[B58] Sprent J. (2005). West African Legumes: the role of nodulation and nitrogen fixation. New Phytol. 168 (1), 326–330. doi: 10.1111/j.1469-8137.2005.01499.x 15998387

[B59] Thirkell T. J. Charters M. D. Elliott A. J. Sait S. M. Field K. J. (2017). Are mycorrhizal fungi our sustainable saviours? Considerations for achieving food security. J. Ecol. 105 (4), 921–929. doi: 10.1111/1365-2745.12788

[B60] Tobar R. Azcón R. Barea J. (1994). Improved nitrogen uptake and transport from ^15^N-labelled nitrate by external hyphae of arbuscular mycorrhiza under water-stressed conditions. New Phytol. 126 (1), 119–122. doi: 10.1111/j.1469-8137.1994.tb07536.x

[B61] Verzeaux J. Hirel B. Dubois F. Lea P. J. Tétu T. (2017). Agricultural practices to improve nitrogen use efficiency through the use of arbuscular mycorrhizae: Basic and agronomic aspects. Plant Sci. 264, 48–56. doi: 10.1016/j.plantsci.2017.08.004 28969802

[B62] Wahbi S. Maghraoui T. Hafidi M. Sanguin H. Oufdou K. Prin Y.. (2016). Enhanced transfer of biologically fixed n from faba bean to intercropped wheat through mycorrhizal symbiosis. Appl. Soil Ecol. 107, 91–98. doi: 10.1016/j.apsoil.2016.05.008

[B63] Wang S. Chen A. Xie K. Yang X. Luo Z. Chen J.. (2020). Functional analysis of the OsNPF4.5 nitrate transporter reveals a conserved mycorrhizal pathway of nitrogen acquisition in plants. Proc. Natl. Acad. Sci. United. States America 117 (28), 16649–16659. doi: 10.1073/pnas.2000926117 PMC7368293 32586957

[B64] Wang G. Sheng L. Zhao D. Sheng J. Wang X. Liao H. (2016). Allocation of nitrogen and carbon is regulated by nodulation and mycorrhizal networks in soybean/maize intercropping system. Front. Plant Sci. 7. doi: 10.3389/fpls.2016.01901 PMC5160927 28018420

[B65] Wang W. Shi J. Xie Q. Jiang Y. Yu N. Wang E. (2017). Nutrient exchange and regulation in arbuscular mycorrhizal symbiosis. Mol. Plant 10 (9), 1147–1158. doi: 10.1016/j.molp.2017.07.012 28782719

[B66] Whiteside M. D. Werner G. D. Caldas V. E. van’t Padje A. Dupin S. E. Elbers B.. (2019). Mycorrhizal fungi respond to resource inequality by moving phosphorus from rich to poor patches across networks. Curr. Biol. 29 (12), 2043–2050. e8. doi: 10.1016/j.cub.2019.04.061 31178314 PMC6584331

[B67] Wipf D. Krajinski F. van Tuinen D. Recorbet G. Courty P. E. (2019). Trading on the arbuscular mycorrhiza market: From arbuscules to common mycorrhizal networks. New Phytol. 223 (3), 1127–1142. doi: 10.1111/nph.15775 30843207

[B68] Xiao H. Ye X. Xu H. Mei Y. Yang Y. Chen X.. (2020). The genetic adaptations of fall armyworm spodoptera frugiperda facilitated its rapid global dispersal and invasion. Mol. Ecol. Resour. 20 (4), 1050–1068. doi: 10.1111/1755-0998.13182 32359007

[B69] Yang S.-Y. Grønlund M. Jakobsen I. Grotemeyer M. S. Rentsch D. Miyao A.. (2012). Nonredundant regulation of rice arbuscular mycorrhizal symbiosis by two members of the PHOSPHATE TRANSPORTER1 gene family. Plant Cell 24 (10), 4236–4251. doi: 10.1105/tpc.112.104901 23073651 PMC3517247

[B70] Yu P. Wang C. Baldauf J. A. Tai H. Gutjahr C. Hochholdinger F.. (2018). Root type and soil phosphate determine the taxonomic landscape of colonizing fungi and the transcriptome of field-grown maize roots. New Phytol. 217 (3), 1240–1253. doi: 10.1111/nph.14893 29154441

[B71] Zhang H. Wang X. Gao Y. Sun B. (2020). Short-term n transfer from alfalfa to maize is dependent more on arbuscular mycorrhizal fungi than root exudates in n deficient soil. Plant Soil 446 (1), 23–41. doi: 10.1007/s11104-019-04333-1

[B72] Zhang Y. C. Zou Y. N. Liu L. P. Wu Q. S. (2019). Common mycorrhizal networks activate salicylic acid defense responses of trifoliate orange (*Poncirus trifoliata*). J. Integr. Plant Biol. 61 (10), 1099–1111. doi: 10.1111/jipb.12743 30450833

[B73] Züst T. Agrawal A. A. (2017). Trade-offs between plant growth and defense against insect herbivory: An emerging mechanistic synthesis. Annu. Rev. Plant Biol. 68, 513–534. doi: 10.1146/annurev-arplant-042916-040856 28142282

