# Supplementary material for: Aboveground herbivory does not affect mycorrhiza-dependent nitrogen acquisition from soil but inhibits mycorrhizal network-mediated nitrogen interplant transfer in maize
Source: Front Plant Sci. 2022 Dec 14;13:1080416. doi: 10.3389/fpls.2022.1080416 (PMC9795027; doi:10.3389/fpls.2022.1080416)
Supplement: Supplementary file 1 [file DataSheet_1.pdf]

Table S1. Gene-specific primers for quantitative RT-PCR

Gene	Gene ID	Specific Primer
ZmGAPDH	*Zm00001eb173410*	F:5’-AGCAGGTCGAGCATCTTCG-3<br>R:5’-CTGTAGCCCCACTCGTTGTC-3’
ZmPht1;6	*Zm00001eb359490*	F:5’-GATCACGCCTCGAACGATCT-3’<br>R:5’-ATGCATACGGTGTATGGCCC-3’
ZmAMT3;1	*Zm00001eb366590*	F: 5'-TCGTCACCCATCAGTGGCAG-3'<br>R: 5'-ACAGCCACGACGTCAAACAC-3'
ZmAMT4;1	*Zm00001e012038*	F:5’-TCTCCTATACCATCGGGGCG-3'<br>R:5’-CGGAGGAAAGGGTGGATGACG-3'
ZmNRT2;3	*Zm00001e028139*	F:5’-CAGTCCAAGCACACAGTCCC-3'<br>R:5’-AGTTACCACACGGATGCACA-3'

Figure S1.

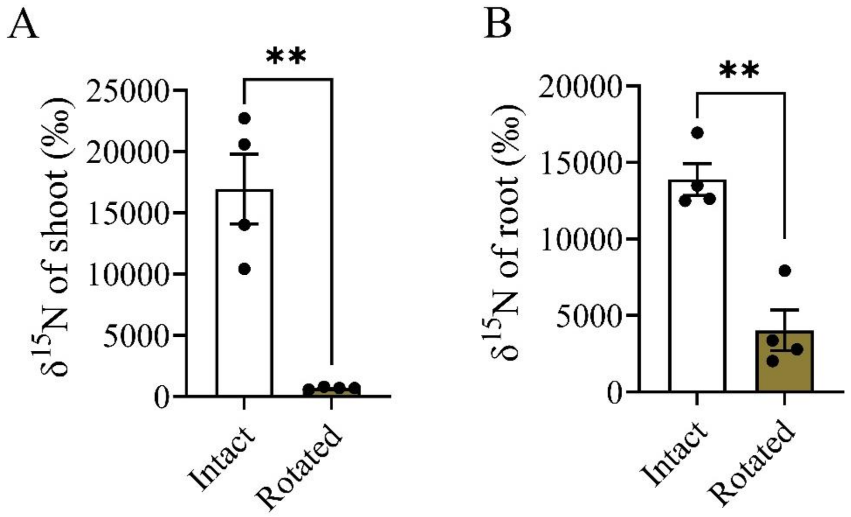

Figure S1. Mycorrhizal symbiosis promoted the δ^{15}N in shoot (A) and root (B) of maize plant. Maize plants were inoculated with *Rir* and were cultivated in boxes with a PVC core in each box as illustrated in experiment II. Five weeks post *Rir* inoculation, the PVC core was injected with $(^{15}NH_4)_2SO_4$ solution, and was then either rotated daily (for the purpose of damaging the mycelium) or untreated (Intact). Plants were harvested to measure ^{15}N which represented as the form of isotopic ratio. Data are means ± SE of four biological replicates. Asterisks indicate significant differences (student's t-test, $**P < 0.01$).

Figure S2.

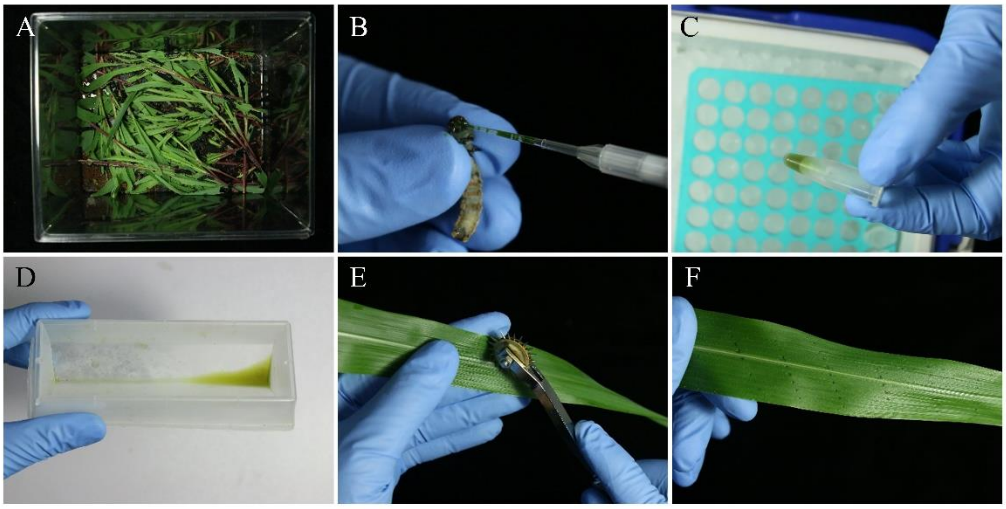

Figure S2. The procedures of insect oral secretion collection and wounding + oral secretion (W+OS). The oral secretion (OS) was collected from the 5th instar *Spodoptera frugiperda* larvae fed on maize seedlings (A). The OS collection process is briefly described below: gently grasped the 5th instar larvae of *Spodoptera frugiperda* between the thumb and index finger, and touched the larvae's mouth with a 0.1-10 μL pipette tip to stimulate the larvae to spit out oral secretions (B). The oral secretions of the larvae were quickly pipetted and collected into pre-chilled 1.5 mL EP tubes (C). Centrifuge collected secretions at 12,000 rpm for 10 min at 4°C to remove food debris; oral secretions were finally diluted 5-fold with ddH_2O before use (D). Mechanical damage and *Spodoptera frugiperda* saliva treatment (W+OS): the maize seedlings with the same growth phenotype were selected for the W+OS treatment, a barbed roller dipped with the OS was used to pierce each leaf of the maize plants to imitate the insect herbivore (E). The number of piercing holes was determined according to the size of the leaf, generally 50-100 holes/leaf (F). The treatment was repeated every 3 days, 3 times during the test period, and the samples were collected after 10 days of treatment.

Figure S3.

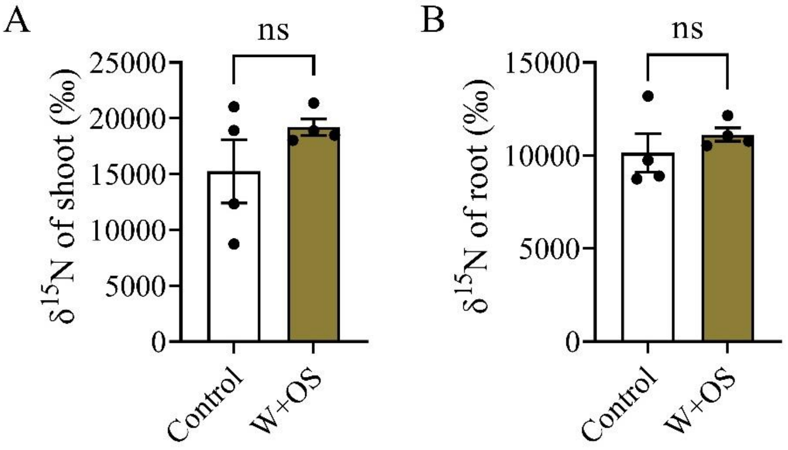

Figure S3. Herbivory did not affect the δ^{15}N in shoot (A) and root (B) of maize plant. Maize plants were inoculated with *Rir* and were cultivated in self-designed boxes, with a PVC core in each box as illustrated in experiment III. Five weeks post *Rir* inoculation, the PVC core was injected with $(^{15}NH_4)_2SO_4$ solution, and was then either rotated daily (for the purpose of damaging the mycelium) or untreated (static) for 10 days, during which W+OS treatment was introduced. Plants were then harvested to analyze δ^{15}N. Data are means ± SE of four biological replicates. Asterisks indicate significant differences (student's t-test; ns, no significance).

Figure S4.

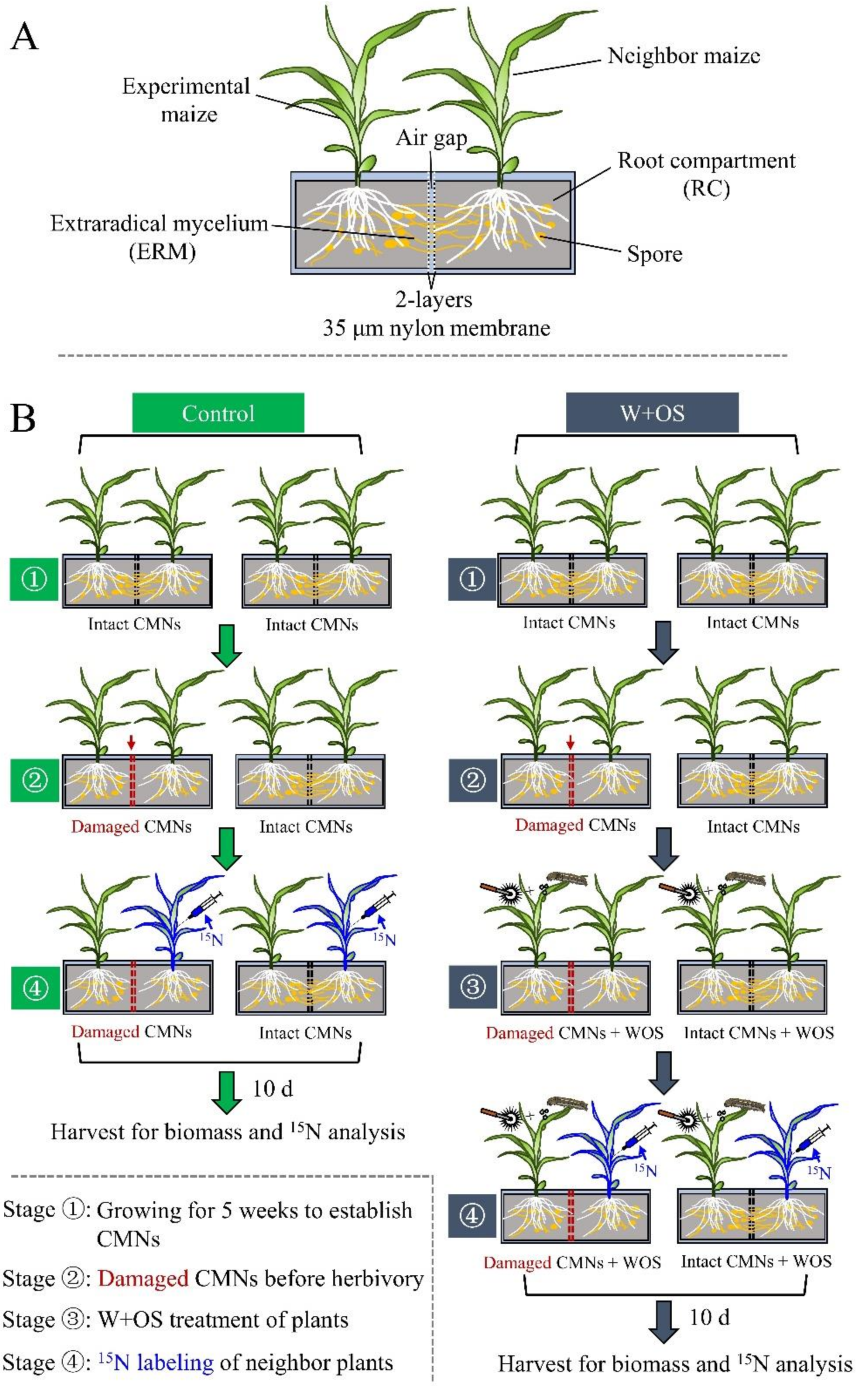

Figure S4. The flowchart for detection of N transfer between maize plants in Experiment IV. (A) Mesocosm designed for maize plants to form common mycorrhizal networks (CMNs). (B) The flowchart for detection of N transfer mediated by CMNs between two maize plants without W+OS treatment (Control) and in the presence of W+OS treatment (W+OS).

Figure S5.

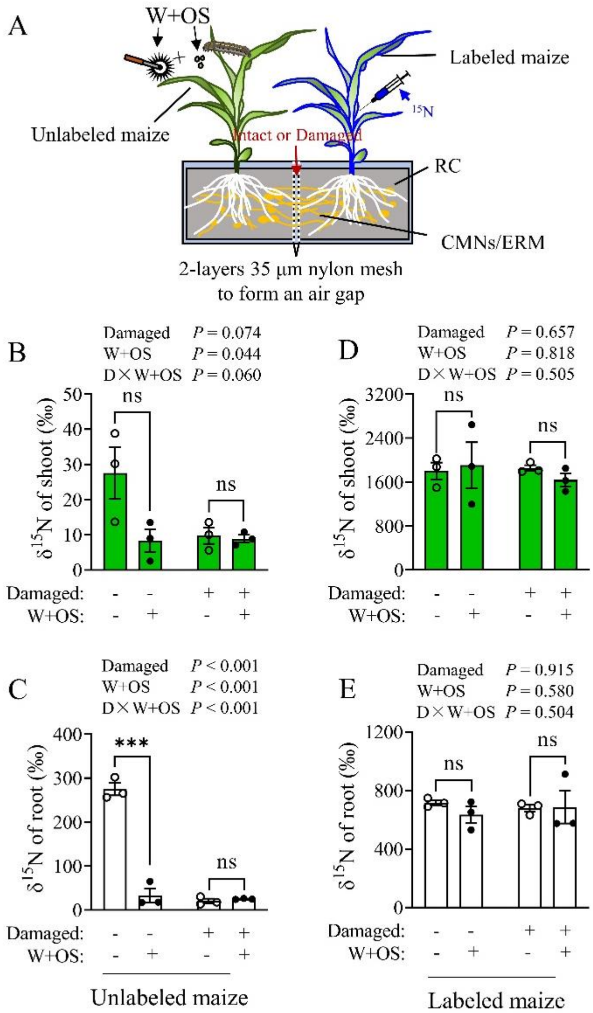

Figure S5. The maize plant obtained N that transferred from its neighboring plant. Maize plants were inoculated with *Rir* and were cultivated in self-designed mesocosms as demonstrated in experiment IV (A) for five weeks, RC, root compartment; CMNs, common mycorrhizal networks; ERM, extraradical mycorrhizal mycelia. The CMNs established between two maize plants were either damaged daily or intact. ten days after ^{15}N labeling and W+OS treatment, **δ**^{15}N was detected in unlabeled plants (B and C) and ^{15}N labeled plant (D and E). Data are means ± SE of three biological replicates. Asterisks indicate significant differences (student's t-test, ***P < 0.001; ns, no significance).

Figure S6.

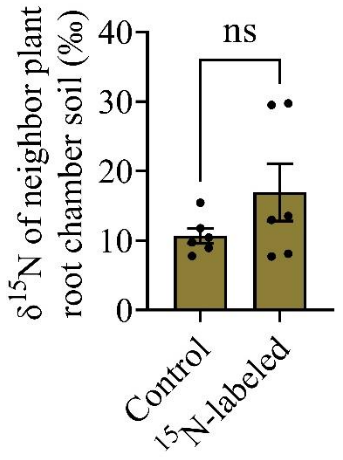

Figure S6. The labeled ^{15}N excreted from the roots of maize plants. Maize plants were each cultivated in a box without *Rir* inoculation. Five weeks later, these plants were either injected with ($^{15}NH_4$)$_2SO_4$ (^{15}N-labeled) solution or untreated (Control). The treatment of ^{15}N-labeling was repeated every 3 days, and 10 days later, the soil in the box of the Control group and ^{15}N-labeled group were collected for analysis of $\delta^{15}N$. Data are means ± SE of six biological replicates. Asterisks indicate significant differences (student's t-test; ns, no significance).

Figure S7.

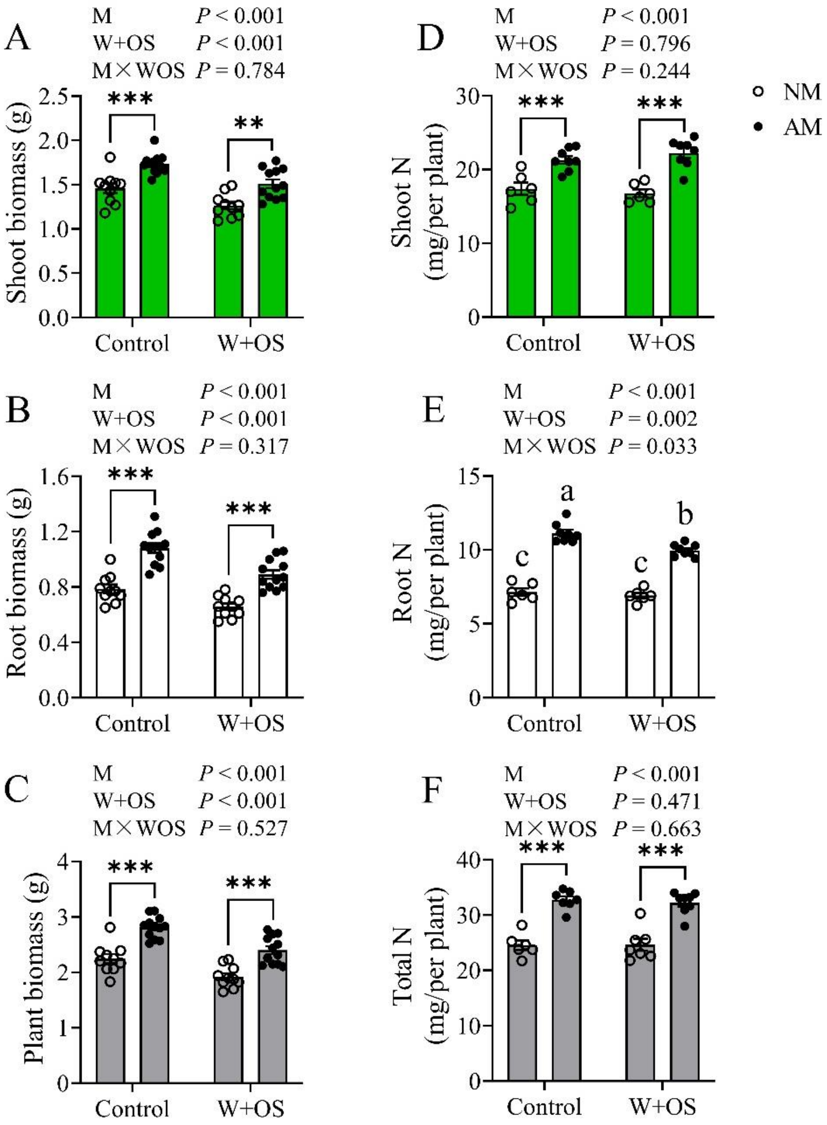

Figure S7. The effect of aboveground herbivory and mycorrhizal symbiosis on plant biomass weight and nitrogen content of maize plants. After 5-week cultivation, leaves of non-mycorrhizal (NM) and arbuscular mycorrhizal (AM) maize plant were either untreated (control) or treated with oral secretion of *Spodoptera frugiperda* larvae in wounded sites (W+OS) every 3 days. 10 days later, plants were harvested for the analysis of biomass (A-C), and nitrogen content (D-F); for analysis of biomass, n = 10-12, for the analysis of nitrogen content, n = 6-8. Data are means ± SE of biological replicates. Interactions among M (Mycorrhizal) × W+OS were analyzed by two-way ANOVA. Letters above bars indicate significant differences among treatments ($P < 0.05$ according to Tukey's post hoc test). Asterisks indicate significant differences (student's t-test; $^{**}P < 0.01$; $^{***}P < 0.001$; ns, no significance).

Figure S8.

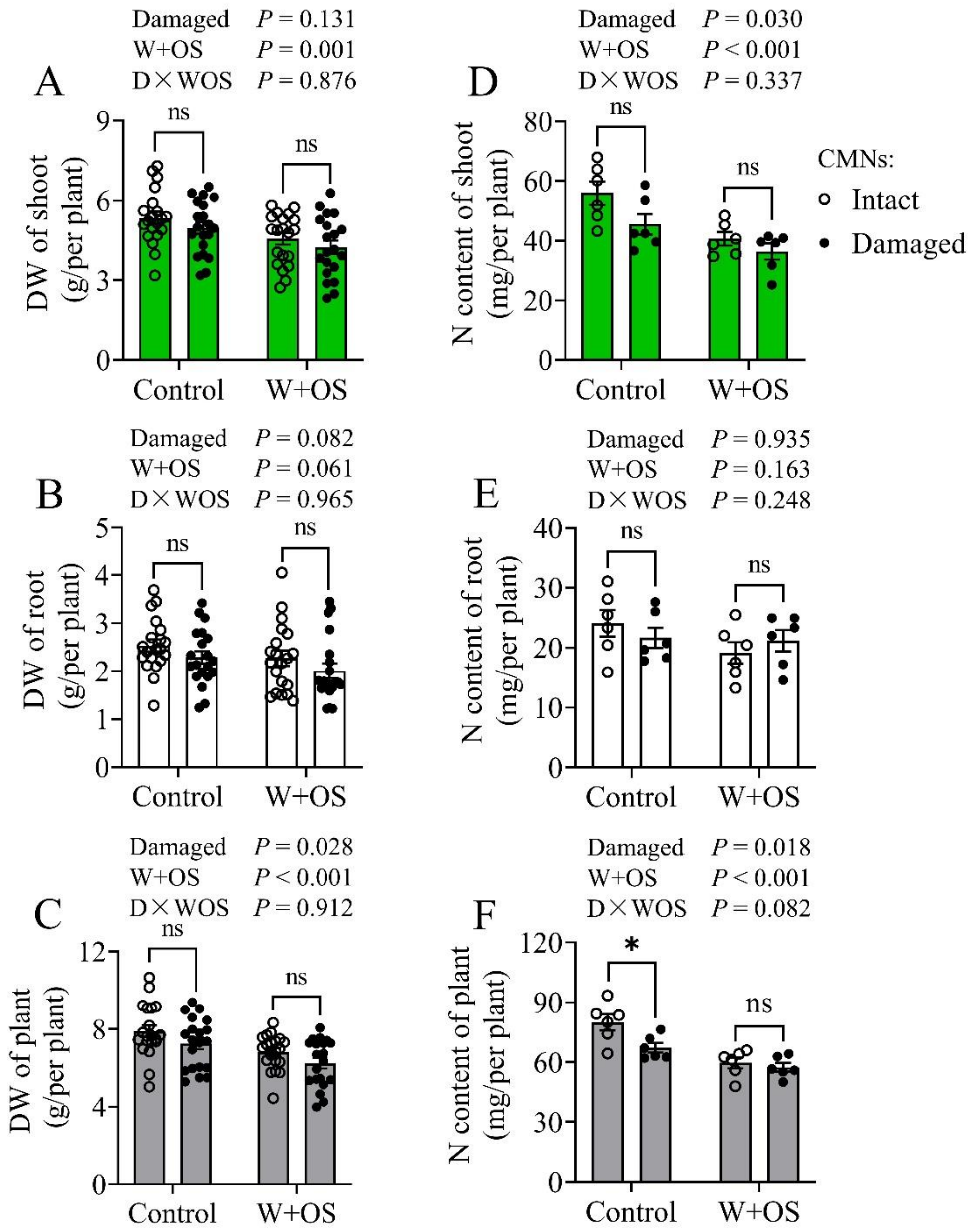

Figure S8. The effect of aboveground herbivory and CMNs connection on plant dry weight and nitrogen content of maize plants. Maize plants were inoculated with *Rir* and were cultivated in self-designed mesocosms as demonstrated in experiment IV. The CMNs established between two maize plants were either damaged daily or intact. ^{15}N was labeled in one of two maize plants in the system for 10 days, during which herbivory treatment was introduced in the unlabeled plant. plants were harvested for the analysis of dry weight (A-C), and nitrogen content (D-F); for analysis of weight, n = 10-12, for analysis of nitrogen content, n = 6-8. Data are means ± SE of biological replicates. Interactions among S (Severed) × H (Herbivory) were analyzed by two-way ANOVA. Letters above bars indicate significant differences among treatments ($P < 0.05$ according to Tukey's post hoc test). Asterisks indicate significant differences (student's t-test, *$P < 0.05$; ns, no significance).
